# Adaptive genetic variation distinguishes Chilean blue mussels (*Mytilus chilensis*) from different marine environments

**DOI:** 10.1002/ece3.2110

**Published:** 2016-04-26

**Authors:** Cristián Araneda, María Angélica Larraín, Benjamin Hecht, Shawn Narum

**Affiliations:** ^1^ Departamento de Producción Animal Facultad de Ciencias Agronómicas Universidad de Chile Avda Santa Rosa 11315, La Pintana 8820808 Santiago Chile; ^2^ Departamento de Ciencia de los Alimentos y Tecnología Química Facultad de Ciencias Químicas y Farmacéuticas Universidad de Chile Sergio Livingstone 1007, Independencia 8380492 Santiago Chile; ^3^ Columbia River Inter‐Tribal Fish Commission 3059‐F National Fish Hatchery Road Hagerman ID 83332

**Keywords:** assignment tests, FST outlier, population structure, RAD‐seq, SNP discovery

## Abstract

Chilean mussel populations have been thought to be panmictic with limited genetic structure. Genotyping‐by‐sequencing approaches have enabled investigation of genomewide variation that may better distinguish populations that have evolved in different environments. We investigated neutral and adaptive genetic variation in *Mytilus* from six locations in southern Chile with 1240 SNPs obtained with RAD‐seq. Differentiation among locations with 891 neutral SNPs was low (*F*_ST_ = 0.005). Higher differentiation was obtained with a panel of 58 putative outlier SNPs (*F*_ST_ = 0.114) indicating the potential for local adaptation. This panel identified clusters of genetically related individuals and demonstrated that much of the differentiation (~92%) could be attributed to the three major regions and environments: extreme conditions in Patagonia, inner bay influenced by aquaculture (Reloncaví), and outer bay (Chiloé Island). Patagonia samples were most distinct, but additional analysis carried out excluding this collection also revealed adaptive divergence between inner and outer bay samples. The four locations within Reloncaví area were most similar with all panels of markers, likely due to similar environments, high gene flow by aquaculture practices, and low geographical distance. Our results and the SNP markers developed will be a powerful tool supporting management and programs of this harvested species.

## Introduction

Genetic data have been widely used in recent years to facilitate delineation of management units; however, this has been difficult for species with high gene flow and low differentiation (Waples 1998). Most marine species of fish and invertebrates have historically been thought to be panmictic with limited genetic structure due to pelagic larvae that are distributed over wide geographical areas by ocean currents (e.g., Grosberg and Cunningham 2001). Thus, it has been particularly challenging to identify management units for marine species (Allendorf et al. 2010), but recent studies have demonstrated the importance of studying genomewide variation for improving estimates of fine‐scale population structure (Sanford and Kelly 2011; Limborg et al. 2012; Milano et al. 2014).

Recent advances in sequencing technology have possible to perform population genetic analyses in nonmodel species using thousands of SNP markers obtained by genotyping by sequencing (GBS) of reduced representation libraries like restriction‐site‐associated DNA (RAD‐seq; Baird et al. 2008). Large panels of SNP markers allow researchers to test for patterns of neutral and adaptive genetic variation related to population structure and local adaptation. High *F*_ST_ loci that are putative outliers have also been shown to be highly informative to determine the geographical origin of individuals (Ogden 2011; Ogden and Linacre 2015). This approach has been useful to improve resolution of fine‐scale population structure of various nonmodel species (Larson et al. 2014) and provide insight into life‐history variation within species (Hess et al. 2013; Pujolar et al. 2014).

Marine mussels of the genus *Mytilus* are widely distributed throughout all the oceans in the world and are typically found in cold and temperate waters of both hemispheres following an antitropical distribution pattern (Gérard et al. 2008). Like many marine invertebrates, mussel larvae have a planktonic lifetime of weeks or months, and thereby can be potentially dispersed over large geographical areas by marine currents or human‐mediated activities (Hilbish et al. 2002). Due to external fertilization, hybrid zones are often observed when different species of mussels inhabit the same geographical area (Hilbish et al. 2002; Toro et al. 2002, 2004a). In southern Chile, Toro et al. (2005) detected the presence of alleles from *M. galloprovincialis* and *M. edulis* using the Me 15‐16 nuclear marker developed by Inoue et al. (1995). However, they did not perform the subsequent restriction analysis developed by Santaclara et al. (2006) to discriminate the allele of *M. galloprovincialis* from *M. chilensis*, confounding both species and *consequently* overestimating the frequency of *M. galloprovincialis* allele in southern Chile. Currently, it is generally accepted that the presence of *M. galloprovincialis* in Chile is restricted to the Arauco Gulf (S 37°06′16″, W 73°21′33″) (Tarifeño et al. 2012). Additionally, *M. trossulus* alleles were first observed in Chile by Larraín et al. (2012), and a subsequent study reported in a hybrid zone of *M. chilensis, M. edulis,* and *M. trossulus* in the Magellan Strait surrounding the international port of Punta Arenas (Oyarzún et al. 2016).

Chilean blue mussel (*M. chilensis*) is an important commercial species in Chile, and it is distributed along approximately 1900 km of the Chilean coast from the Arauco Gulf (35°S) in the north to Cape Horn (55°S) in the south (Hernández and González 1976). Genetic diversity and population structure of this economically important species are unresolved, but critical for supporting management and conservation programs of this harvested species. Genetic structure of *M. chilensis* has been explored using five RAPD primers (54 presumptive dominant loci), and with seven and 26 allozyme loci, in populations from Arauco (35°S) to Punta Arenas (53°S) (Toro et al. 2004b, 2006; Cárcamo et al. 2005), finding no evidence of discrete stocks (0.011 ≤ *F*_ST_ ≤ 0.055), with the possible exception of an austral population from Punta Arenas (53°S). Studies with microsatellite markers have found similar results of limited genetic structure (Larraín et al. 2014, 2015). All of this evidence indicates low genetic differentiation among populations of Chilean blue mussel, likely due to dispersion of long‐lived (45 days) planktotrophic larvae across the Chilean coastline by coastal currents that homogenize populations (Toro et al. 2006).

In this study, we used RAD‐seq to genotype 1240 SNPs in 190 *M. chilensis* individuals collected from six locations in southern Chile, including areas used as seed collection centers for the local mussel aquaculture industry. This study was designed to investigate patterns of neutral versus adaptive genetic variation within this species and identify a subset of genetic markers that could improve the ability to trace individuals to their population of origin, especially in areas with strong aquaculture activities.

## Materials and Methods

### Samples collection and preparation

**Figure 1:**
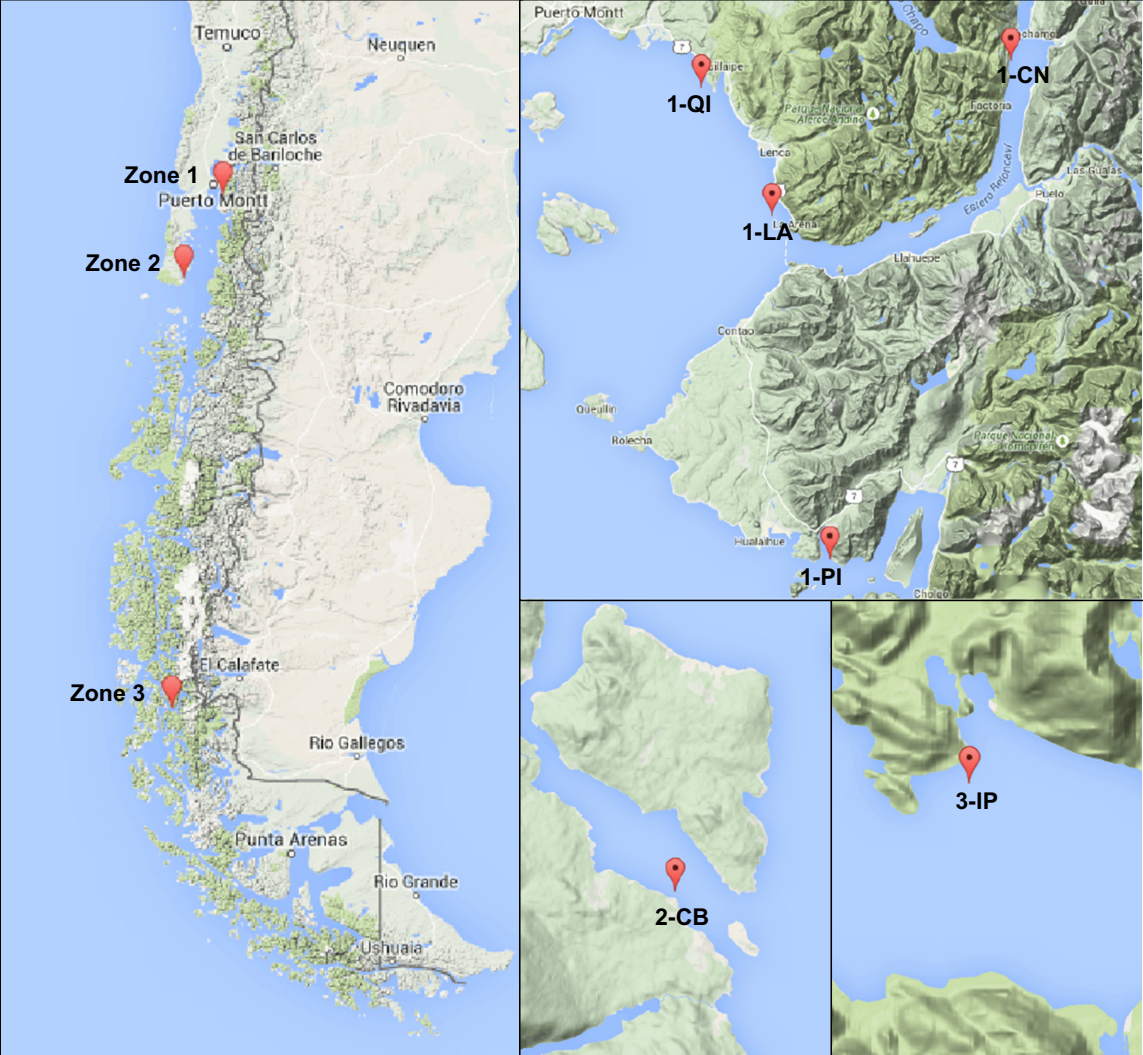
Sampling locations in southern Chile. Codes and geographical positions are indicated in Table 1.

Samples of *M. chilensis* were collected in 2009 from subtidal zones in six different locations, to capture diversity of the southern distribution of Chilean blue mussels in Chile, especially in areas used as seed collection centers for the local aquaculture industry (Table 1 and Fig. 1). For mussel species, “seeds” refer to individuals in the juvenile phase of their life cycle. We included samples from four locations in the Reloncaví Gulf (Quillaipe, Caleta La Arena, Canutillar, and Pichicolo; Fig. 1), one location from southern area of Chiloé Island (Canal Coldita) and one population from the southern Patagonia area (Isla Peel; previously identified to be most genetically differentiated from the others) (Toro et al. 2004b, 2006). All samples were seeds with a shell size of 15–25 mm with the exception of Patagonia samples, which were adults. Mantel tissue was collected from all individuals and stored in ethanol.

**Table 1:** Description of locations, zones, scenarios, number of individual included in the training set (*N*_ts_) and holdout set (*N*_hos_), and overall sample sizes (*N*) analyzed in this study

Code	Sample location	Zone	South latitude/West longitude	Scenarios	*N*_ts_	*N*_hos_	*N*
1‐QI	Quillaipe	1 Reloncaví Gulf	41° 32′ 55,35″/72° 46′ 14,35″	1, 2	15	15	30
1‐PI	Pichicolo	1 Reloncaví Gulf	42° 02′ 23,76″/72° 35′ 27,17′'	1, 2	13	12	25
1‐LA	Caleta La Arena	1 Reloncaví Gulf	41° 41′ 00,00″/72° 40′ 18,92′'	1, 2	20	19	39
1‐CN	Canutillar	1 Reloncaví Gulf	41° 31′ 13,90″/72° 20′ 15,69′'	1, 2	17	16	33
2‐CB	Canal Coldita	2 Chiloé Island	43° 14′ 48,82″/73° 41′ 42,77′'	1, 2	16	15	31
3‐IP	Isla Peel	3 Patagonia	50° 50′ 29,83″/74° 00′ 41,27′'	1	16	16	32

### DNA extraction and species identification

Mantle edge tissue (50–100 mg) was used to extract DNA using the phenol–chloroform method described by Larraín et al. (2012). Extracted DNA was quantified using the Quant‐iT dsDNA pico‐green assay kit (Thermo Fisher Scientific Inc, Waltham, MA) in 96‐well plates and a Perkin Elmer Victor 5 plate reader. Since some of the collected individuals were too small (15–25 mm) to identify phenotypic species, these samples were tested with diagnostic species markers as described by Larraín et al. (2012). In brief, primers Me15‐16 produce different sizes of amplicons by species: 180 pb for *M. edulis*, 168 pb *M. trosuluss,* and 126 pb for *M. chilensis* and *M. galloprovincialis*, posterior digestion with the enzyme *Aci*I cut the *M. galloprovincialis* amplicon while the *M. chilensis* amplicon remains uncut (Santaclara et al. 2006). Three specimens (1.6% of samples) were identified as hybrids and were removed from the study.

### Prediction of expected number of RADtags

Number of RADtag was predicted using the R package SimRAD (Lepais and Weir 2014) using the haploid nuclear DNA content (*C*‐value) published for *M. galloprovincialis* of 1.92 pg (Rodríguez‐Juíz et al. 1996) equivalent to an haploid genome size of 1877.76 Mb. We estimated CG content to be 0.3297 from the analysis of 3.8 Mb of sequence from Araneda et al. (2015).

### RAD library preparation and sequencing

Restriction‐site‐associated DNA (RAD) libraries were prepared with the restriction enzyme *Sbf*I following the methods of Hess et al. (2013) and sequenced on an Illumina^®^ HiSeq 1500 genetic analyzer (Illumina Inc., San Diego, CA). Two libraries were constructed containing 96 individuals per library and 500 ng DNA per individual. In each library, pooled individuals were tagged by ligation of one of 96 unique 6‐bp barcode adapter (P1 adapter) to the *Sbf*I site, and 4 *μ*g was sheared by sonication using a Bioruptor UCD‐300 instrument (Diagenode^®^, Denville, NJ). After sonication, each library was concentrated using the Qiagen MinElute PCR purification kit (Qiagen^®^, Venlo, Netherlands) and submitted to AMPure bead purification and size selection (Agencourt^®^, Beckman Coulter, Brea, CA). Size selection was performed on each library by first binding DNA fragments larger than 700 bp with a 2:1 ratio of DNA to beads, followed by a 1:1 ratio of DNA to suspension buffer (binding DNA fragments larger than 200 bp) in order to yield final products with DNA fragments ranging from 200 to 700 bp. Prior to sequencing, RADtag libraries were quantified by quantitative PCR (Kappa Biosystems Inc, Woburn, MA) on an ABI 7900HT Sequence Detection System (Thermo Fisher Scientific Inc, Waltham, MA). The libraries were then sequenced in a total of four lanes (two each) by single‐end 100 base reads using an Illumina^®^ HiSeq 1500 sequencer (Illumina Inc., San Diego, CA). The total number of reads obtained was 822,488,647, of which 738,252,110 passed the quality filters and were retained for SNP identification (89.76%). The number of sequences obtained per individual ranged from 155,007 to 21,512,094 with an average of 4,236,976 reads. Two individuals from Pichicolo were excluded from subsequent analysis due to low number of quality reads (<807,570).

### Bioinformatics

Software package, version 1.08 (Catchen et al. 2011, 2013), was used for SNP discovery and genotyping. All 100‐bp reads were quality‐filtered (Phred 33), trimmed to 75 bp at the 3′ end, and demultiplexed based on barcodes using “process_radtags” in STACKS. Prior to running “ustacks” for SNP discovery, all samples were standardized to 3.5 million reads to assure that each individual was contributing in same proportion of the overall variation in sequence (Hecht et al. 2013). Discovery of SNPs with the “ustacks” module included setting the “m” parameter to 10 (minimum depth of coverage required to create a stack) and using the “snp model type.” A catalog of SNPs was created with the “cstacks” module including two individuals from every location, and this catalog was used to genotype each individual with the “sstacks” module. Finally, the “population” module was used to produce a single Genepop formatted file containing 190 individuals from six populations.

Several quality filters were applied during the SNP discovery process in order to remove putative false SNPs and paralogs. First, SNPs with genotyping success lower than 70% and minor allele frequency (MAF) less than 5% were removed, retaining 4305 SNPs for further steps. Second, 2140 SNPs were removed because they were not genotyped in all six populations. Third, only one bi‐allelic SNP per RADtag was permitted, removing another 865 SNPs. Fourth, to filter out potential paralogous markers, 60 SNPs were removed because they showed significant deviations from Hardy–Weinberg equilibrium (HWE; Genepop 4.2; Raymond and Rousset 1995; Rousset 2008) in three or more locations with FDR‐BY corrected of critical level of 0.005238 (Narum 2006). This combination of filters resulted in a remaining panel of 1240 SNPs genotyped on 190 individuals.

### Detection of loci under positive selection and neutral loci

Two scenarios using LOSITAN (Antao et al. 2008) were analyzed to produce different sets of candidate SNPs. Scenario 1 was performed including all six locations and scenario 2 excluded the most divergent collection (Isla Peel samples from Patagonia zone) and keeping only five locations in the geographical area used by the Chilean aquaculture industry as a collection site (Fig. 1, Table 1). In both scenarios, LOSITAN was run using FDIST2 method (Beaumont and Nichols 1996) with 1,000,000 simulations and confidence interval (CI) 0.995, false discovery rate of 0.1, and subsample size of 50. Simulated neutral *F*_ST_ values were 0.01166 and 0.00597 for scenarios 1 and 2, respectively. In order to reduce false positives, loci were considered candidates under positive selection above a probability level of 0.995, and neutral loci were defined as falling between intervals of 10–90% of the *F*_ST_ distribution. Underlying population structure was not considered to be a substantial factor since global *F*_ST_ was near zero across all loci (*F*_ST_ = 0.005).

### Population structure and assignment tests

Population analyses were performed independently with three panels of markers: putatively neutral loci and two panels of outlier loci obtained from scenarios 1 and 2. A discriminant analysis of principal component (DAPC) using the R package adegenet 3.1‐1 (Jombart 2008; Jombart and Ahmed 2011) was performed and the number of clusters (K) was identified using the lowest Bayesian information criteria (BIC). A cluster is an abstract object composed by genetically similar individuals that are not necessarily coincident with collecting locations. Global and pairwise *F*_ST_ values (Weir and Cockerham 1984) were computed with Genepop 4.2. (Raymond and Rousset 1995; Rousset 2008), and 95% confidence intervals were estimated by bootstrapping using the R package diveRsity with 10,000 replicates (Keenan et al. 2013).

Isolation‐by‐distance (IBD) gene flow analysis using a Mantel test (Rousset 1997) was performed on *F*_ST_/(1−*F*_ST_) and the logarithm of geographical distance estimated following the coastline among pairs of locations, with 10,000 permutations in program ISOLDE implemented in Genepop on the web (http://genepop.curtin.edu.au/index.html).

Assignment power of SNP panels was evaluated with leave‐one‐out tests (LOO) and with the double cross‐validation proposed by Anderson (2010) using the “training set/holdout set” protocol (STH) and implemented in GeneClass2.0 (Piry et al. 2004), to compare the performance of both outlier SNP panels to resolve fine‐scale population structure. With the LOO method, individuals were assigned to a population if the assignment probability to that population was higher than to any other population. We selected the frequency‐based algorithm (Paetkau et al. 1995) as recommended by Piry et al. (2004) and because a former study showed it had the best performance in matching *Mytilus* individuals to their geographical origin (Larraín et al. 2014). Additionally, to avoid high‐grade bias in estimating classification accuracy (Anderson 2010), we randomly divided the samples into training and holdout sets (Table 1). The training set was used as baseline to reassign individuals from the holdout set (Paetkau et al. 1995).

## Results

The number of RADtags predicted using SimRAD was 8530 close to the mean number of RADtags obtained per samples with STACKS (10,030). In these RADtags initially were included 16,888 presumptive SNPs in the catalog, in which 4305 SNPs were genotyped in the 70% or more samples. However, after subsequent filtering, a final panel of 1240 SNPs was retained with an average genotyping success of 90% in the remaining 190 samples.

### Genetic diversity

Average expected heterozygosities per location across all 1240 SNPs were similar, with values of 0.2321 for Caleta La Arena, 0.2322 for Quillaipe, 0.2330 for Canutillar, and 0.2333 for Pichicolo in the Reloncaví zone, 0.2328 for Canal Coldita in Chiloé Island zone, and 0.2244 for Isla Peel in Patagonia zone.

### Detection of loci under positive selection and neutral loci

Outlier analyses run in LOSITAN identified 58 and 34 SNPs as candidates for positive selection among scenarios 1 and 2, respectively (upper limit of *F*_ST_ CI 99.5%), with 17 shared SNPs between both scenarios. Outlier tests also identified 981 putatively neutral loci for scenario 1, which fell inside of a conservative CI of 10–90% of *F*_ST_ distribution.

### Population structure

Global *F*_ST_ of the neutral panel in scenario 1 was very low (0.005) but significant (95% CI: 0.001, 0.011). Higher global genetic differentiation was observed in scenario 2 with a *F*_ST_ of 0.088 (95% CI: 0.065, 0.111) with the panel of 34 SNPs and for scenario 1 with a global *F*_ST_ of 0.114 (95% CI: 0.101, 0.128) for the 58 SNP panel.

Pairwise *F*_ST_ values for the neutral panel were not significantly different from zero for 10 of the 15 interlocation comparisons as shown by 95% confidence intervals, and significant values were only observed in comparisons where Isla Peel location (Patagonia zone) was involved (Table 2). This pattern of high pairwise *F*_ST_ values between Isla Peel and the other five northern locations was observed for scenario 1, with an average pairwise *F*_ST_ value of 0.228 (Table 2).

**Table 2:** Pairwise *F*_ST_ calculated using 981 neutral SNPs (above diagonal) and using 58 outlier SNPs (below diagonal, scenario 1) among six collection sites of *Mytilus chilensis* in southern Chile

	1‐QI Quillaipe	1‐PI Pichicolo	1‐LA Caleta La Arena	1‐CN Canutillar	2‐CB Canal Coldita	3‐IP Isla Peel
1‐QI		−0.004 (−0.013, 0.008)	0.001 (−0.007, 0.010)	−0.001 (−0.008, 0.010)	0.004 (−0.005, 0.015)	0.013 (0.004, 0.023)
1‐PI	0.008 (−0.010, 0.032)		0.001 (−0.009, 0.012)	−0.003 (−0.012, 0.009)	0.001 (−0.008, 0.013)	0.012 (0.002, 0.024)
1‐LA	0.005 (−0.011, 0.026)	0.023 (0.002, 0.050)		0.001 (−0.006, 0.010)	0.005 (−0.002, 0.015)	0.015 (0.007, 0.024)
1‐CN	0.007 (−0.010, 0.030)	0.008 (−0.012, 0.034)	0.017 (−0.001, 0.044)		0.004 (−0.003, 0.014)	0.013 (0.005, 0.023)
2‐CB	0.080 (0.051, 0.110)	0.060 (0.033, 0.093)	0.101 (0.073, 0.129)	0.082 (0.054, 0.129)		0.012 (0.003, 0.022)
3‐IP	0.228 (0.195, 0.263)	0.217 (0.183, 0.252)	0.248 (0.218, 0.279)	0.231 (0.202, 0.263)	0.216 (0.189, 0.242)	

95% CIs are showed in parentheses.

On the other hand, the location Canal Coldita from Chiloé Island zone shows moderate genetic differentiation with the other four populations from Reloncaví Gulf with an average pairwise *F*_ST_ of 0.081 in scenario 1. A similar pattern was observed for pairwise *F*_ST_ in scenario 2, with significant genetic differentiation observed between Canal Coldita (Chiloé Island zone) and the four populations from Reloncaví zone, but with a higher average pairwise *F*_ST_ than scenario 1 of 0.159.

The four populations from Reloncaví zone showed practically zero genetic differentiation according to the limits of 95% CI for the *F*_ST_ values in both scenarios. However, there were two exceptions with the comparisons between Pichicolo and Caleta La Arena for scenario 1 (*F*_ST_ = 0.023; 95% CI: 0.002, 0.050; Table 2) and scenario 2 (*F*_ST_ = 0.034; 95% CI: 0.004, 0.074; Table 3), and the comparison between Canutillar and Caleta La Arena for the scenario 2 (*F*_ST_ = 0.029; 95% CI: 0.001, 0.065; Table 3).

**Table 3:** Pairwise *F*_ST_ calculated using 34 outlier SNPs under putative directional selection among five collection sites (scenario 2) of *Mytilus chilensis* in southern Chile

	1‐QI Quillaipe	1‐PI Pichicolo	1‐LA Caleta La Arena	1‐CN Canutillar	2‐CB Canal Coldita
1‐QI		0.013 (−0.012, 0.046)	0.017 (−0.009, 0.051)	0.013 (−0.010, 0.043)	0.155 (0.103, 0.208)
1‐PI			0.034 (0.004, 0.074)	0.009 (−0.014, 0.037)	0.118 (0.072, 0.167)
1‐LA				0.029 (0.001, 0.065)	0.198 (0.152, 0,242)
1‐CN					0.164 (0.118, 0.207)

95% CIs are showed in parentheses.

### Assignment to origin populations

The lowest Bayesian information criterion (BIC) on DAPC for the putatively neutral panel was obtained for *K* = 1, indicating only one cluster across the three studied zones when this panel was used. However, more than one cluster was evident for the subsequent scenarios that included putative outlier SNPs. For scenario 1, three clusters (*K* = 3) were identified with DAPC with two discriminant functions extracted with 60 principal components retaining a 93.3% of conserved variance. For scenario 2, again three clusters (*K* = 3) were observed with 50 principal components retaining a 97.8% of conserved variance (Fig. 3).

**Figure 2:**
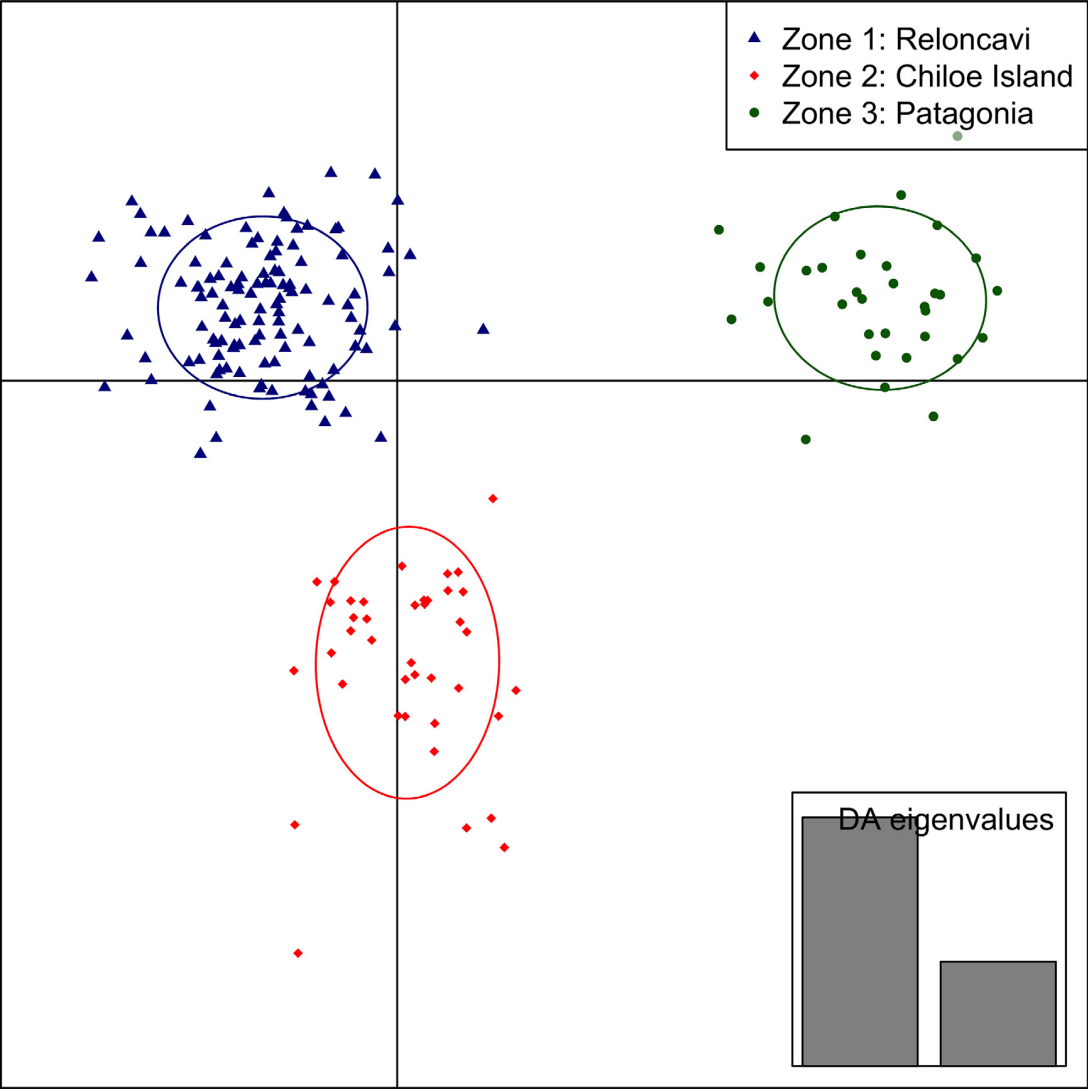
Clusters obtained by discriminant analysis of principal component in scenario 1 (58 putative outlier SNPs and six locations). Clusters are shown by different colors and inertia ellipses, while dots, triangles, and rhombs represent individuals.

The three clusters observed in scenario 1 correspond to the three main geographical zones included in this study (Fig. 2). All individuals (100%) from Isla Peel (Patagonia zone) were assigned to one cluster, 28 of 31 individuals (90.3%) from Canal Coldita (Chiloé Island zone) were assigned to a second cluster, and 115 of 127 individual (90.6%) from the four locations in Reloncaví zone were classified to a third different cluster. The performance of assignment with the panel of 58 SNPs was consistent when it was evaluated with the LOO and STH methods. The correct re‐assignment to Patagonia, Chiloé Island, and Reloncaví zones was 100%, 83.9%, and 92.1% for LOO method and 100%, 73.3%, and 96.8% for STH method, respectively (Table 4).

**Table 4:** Comparison between individual's classifications obtained from discriminant analysis of principal component (DAPC) and assignment of individuals to origin locations using GeneClass2 in *Mytilus chilensis* in southern Chile, scenario 1

Location	DAPC Clusters			Assignment obtained with GeneClass2^a^													
				Leave One Out							Simple Training and Holdout						
				Reloncaví				Chiloé	Patagonia		Reloncaví				Chiloé	Patagonia	
	1	2	3	1‐QI	1‐PI	1‐LA	1‐CN	2‐CB	3‐IP	Total	1‐QI	1‐PI	1‐LA	1‐CN	2‐CB	3‐IP	Total
1‐QI	28	2	0	**7**	2	12	7	2	0	30	**4**	1	7	3	0	0	15
1‐PI	19	6	0	2	**8**	5	6	4	0	25	4	**4**	1	2	1	0	12
1‐LA	38	1	0	11	4	**17**	6	1	0	39	5	0	**11**	3	0	0	19
1‐CN	30	3	0	8	2	7	**13**	3	0	33	4	2	5	**4**	1	0	16
2‐CB	3	28	0	2	1	1	1	**26**	0	31	2	1	0	1	**11**	0	15
3‐IP	0	0	32	0	0	0	0	0	**32**	32	0	0	0	0	0	**16**	16

a Correct assignment to origin location is on diagonal of table in bold font.

Gray cells identify individuals from Reloncaví locations correctly reassigned to Reloncaví zone.

**Figure 3:**
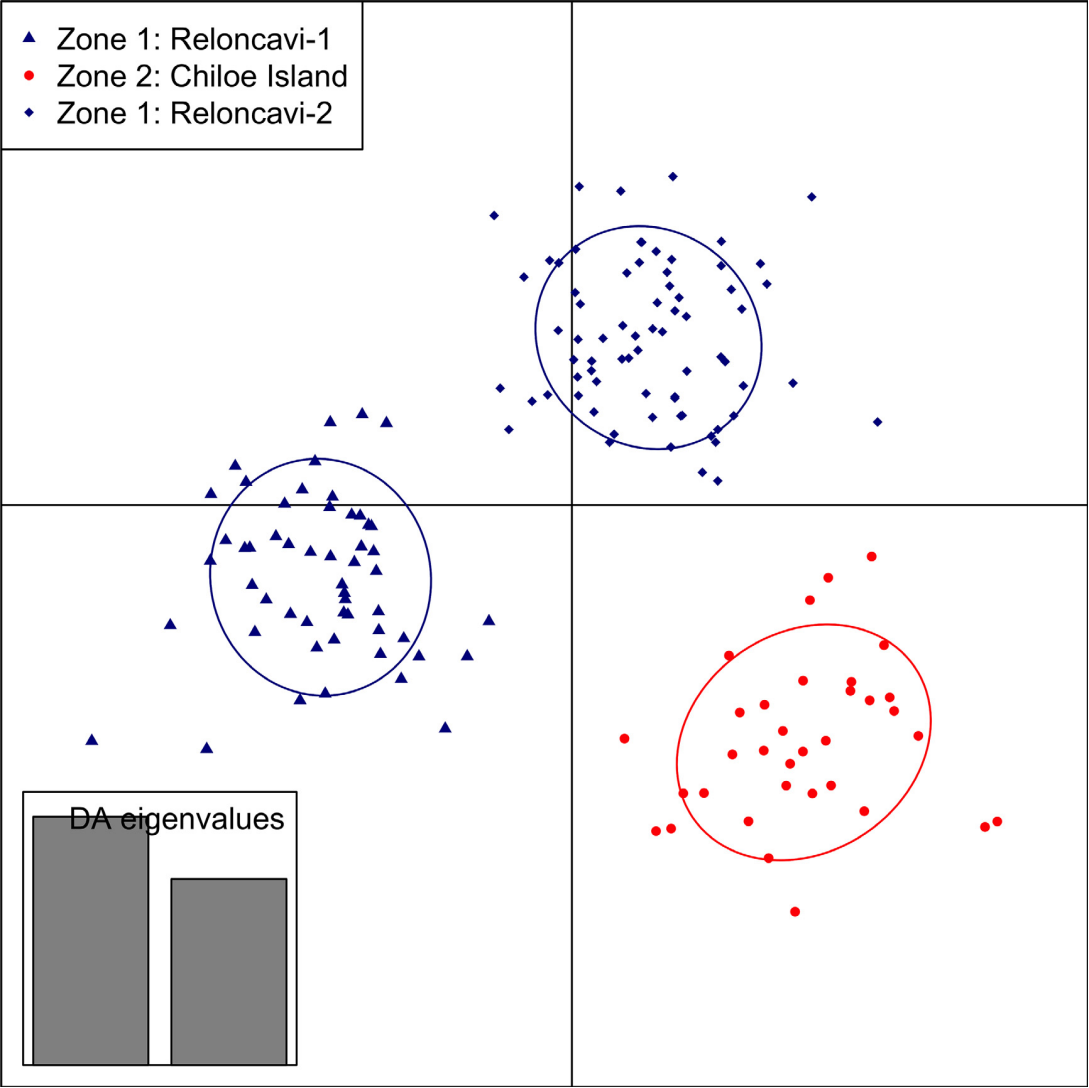
Clusters obtained by discriminant analysis of principal component in scenario 2 data (34 putatively outlier SNPs and five locations). Clusters are shown by different colors and inertia ellipses, while dots, triangles, and rhombs represent individuals.

In scenario 2, DAPC classified 83.9% of samples from Canal Coldita (Chiloé Island zone) in one cluster. Assignment to this location with the panel of 34 SNPs was 87.1% (27 of 31 samples) for LOO method and 86.7% (13 of 15 samples) for STH method (Table 5). Interestingly, individuals from the four locations from Reloncaví zone were classified in two different clusters with DAPC (Fig. 3). However, assignment with GeneClass2 to the Reloncaví zone with this panel of SNPs was high, 94.5% and 93.5% using the LOO and STH methods (Table 5), respectively.

**Table 5:** Comparison between individual's classifications obtained from discriminant analysis of principal component (DAPC) and assignment of individuals to origin locations using GeneClass2 in *Mytilus chilensis* in southern Chile, scenario 2

Location	DAPC Clusters			Assignment obtained with GeneClass2^a^											
				Leave One Out						Simple Training and Holdout					
				Reloncaví				Chiloé		Reloncaví				Chiloé	
	1	2	3	1‐QI	1‐PI	1‐LA	1‐CN	2‐CB	Total	1‐QI	1‐PI	1‐LA	1‐CN	2‐CB	Total
1‐QI	16	12	2	**12**	3	8	6	1	30	**5**	1	6	2	1	15
1‐PI	16	6	3	6	**9**	4	4	2	25	2	**2**	3	3	2	12
1‐LA	17	21	1	9	3	**21**	5	1	39	4	2	**9**	4	0	19
1‐CN	18	13	2	4	7	8	**12**	3	33	4	2	3	**6**	1	16
2‐CB	4	1	26	1	2	1	0	**27**	31	1	0	0	1	**13**	15

a Correct assignment to origin location is on diagonal of table in bold font.

Gray cells identify individuals from Reloncaví locations correctly reassigned to Reloncaví zone.

The IBD analysis showed significant and positive Spearman correlations for the neutral SNP panel (*r*_S _= 0.863, right‐tailed *P* = 0.0263) and outlier SNPs in scenario 1 (*r*_S_ = 0.9393, right‐tailed *P* = 0.0054) since they included the most distinct and geographically distant collection from Patagonia. However, for scenario 2 (excluding the most austral population of Isla Peel), the IBD Spearman correlation was nonsignificant (*r*_S_ = 0.6485 right‐tailed *P* = 0.1491).

## Discussion

Reviews of allozymic and microsatellite studies have revealed lower levels of genetic differentiation and higher genetic diversity among populations of marine fishes in comparison with anadromous and freshwater fishes (Ward et al. 1994; DeWoody and Avise 2000). These differences have been ascribed to high levels of gene flow in marine environment and large effective population sizes (Ward et al. 1994). Panmixia has been proposed for other marine invertebrates with long‐lived planktonic larvae like Chilean blue mussels due to estimates of low *F*_ST_ from allozymes and microsatellite studies (Cárcamo et al. 2005; Toro et al. 2006; Larraín et al. 2014, 2015). However, recent studies with much larger panels of SNP markers have begun to elucidate more extensive patterns of population structure in marine species as a result of sampling both neutral and adaptive loci throughout the genome (Candy et al. 2015).

For *Mytilus* species, few SNP markers have been published and molecular resources are scarce. Previous SNP markers have been developed for pedigree reconstruction to assist breeding programs (Vera et al. 2010; Nguyen et al. 2014; Pino‐Querido et al. 2015), and also for identification of *Mytilus* species or hybridization in Europe (Zbawicka et al. 2012, 2014), but to date no SNPs have been developed specifically to assess population structure of South American populations or taxa. SNP markers in *Mytilus* have been developed by mining EST databases (Vera et al. 2010; Zbawicka et al. 2012), shotgun genome sequencing using Illumina technology (Nguyen et al. 2014), and targeted enrichment sequencing (Fraïsse et al. 2015). However, with declining costs of sequencing technology (Liu et al. 2012) and the use of RAD‐seq to reduce genome complexity (Baird et al. 2008), it is possible to discover and genotype SNP markers at same time in an effective way, opening the opportunity to apply SNPs in nonmodel species (Ogden 2011; Narum et al. 2013). Therefore, SNPs developed by GBS methods such as RAD‐seq or double‐digest RAD‐seq (Peterson et al. 2012) are emerging in the recent literature (Saarman and Pogson 2015). Thus, for the current study, we used RAD‐seq to develop 1240 SNP markers *de novo* and identified outlier loci to effectively characterize the genetic structure of *M. chilensis* from southern Chile, focusing in the area with strong aquaculture activities in order to trace the individuals to their origin populations. With this approach, our study identified finer scale genetic differentiation than has been detected previously for *M. chilensis*. Our analysis of population structure using the neutral panel of 981 SNPs showed nonsignificant *F*_ST_ values for the locations in the area used by Chilean aquaculture industry concordant with the high gene flow within this aquaculture production region due to exchange of seed stocks among facilities (Pantoja et al. 2011). However, the samples from Patagonia zone (Isla Peel) showed low (average pairwise *F*_ST_ = 0.0127) but significant differentiation with the five northern locations, confirming the findings of Toro et al. (2004b, 2006). Further analyses with outlier loci for two scenarios of fine‐scale population structure revealed more extensive genetic differentiation for *M. chilensis* in the study zones.

We analyzed collections of *M. chilensis* from three different local environments: inner protected bays submitted to intense aquaculture (salmon and mussel production) in Reloncaví zone, outer bay exposed to open sea influence in Chiloé Island, and extreme climatic conditions in Patagonia zone (scenario 1) with different contribution of freshwater and variable levels of salinity. Across these three zones, the 58 outlier SNP panel showed the highest levels of genetic differentiation ever reported among collections from these three different zones, and they consistently discriminate individuals collected from these three geographical areas using DAPC, LOO, and STH reallocation methods. This pattern of genetic differentiation was concordant with an effect of isolation by distance at a large geographical scale. Our analyses support strong genetic differentiation between the Patagonia location and northern locations (Reloncaví and Chiloé Island zones). This is consistent with previous studies using RAPD and allozyme markers (Toro et al. 2004b, 2006), and also with our findings with the neutral panel of 981 SNPs. Large differentiation of samples from Isla Peel could be due to a combination of neutral and adaptive processes. Isolating (neutral) effects of the poleward Cape Horn current (Strub et al. 1998) presumably restrict migration of larvae from Patagonia zone to the north. Adaptation to local channel conditions in Patagonia is also possible with high precipitation levels (2.5 m/year), water discharge from rivers and glaciers, extreme photoperiod regime, and very low water temperature (5.5°C at 54°S) in this zone (Pantoja et al. 2011). Samples from the Canal Coldita location (Chiloé Island zone) were also highly distinct from the other two regions with both outlier SNP panels, which have not been previously detected with other molecular markers. This possibly reflects different environmental conditions in the southern area of Chiloé Island influenced by sea water (Strub et al. 1998) with salinity of 31.1 ppt reported in Quellón near the Canal Coldita location which is higher than the Reloncaví zone (average salinity of 21.5 ± 1.55 ppt) (Aranda et al. 2015).

Samples from the Reloncaví zone in the inner sea of Chiloé (41–44°S) were distinct from the other two zones, supporting the finding that the population of *M. chilensis* in Chiloé is genetically divergent with these outlier loci. The four sites within Reloncaví zone were not differentiated with both outlier SNP panels, possibly due to less variable environmental conditions with close levels of salinity ranging from 19.4 ppt in Cochamó (close to Canutillar location) to 21.0 ppt in Quillaipe and similar average water temperature (17.3 ± 0.7°C in summer and 11.4 ± 0.3°C in winter; Aranda et al. 2015). However, Reloncaví samples were grouped in two DAPC clusters with balanced distribution of individuals from each location in each cluster. This pattern could indicate two divergent groups in the Reloncaví zone that could be explained by different larval cohorts representing individuals of different ages. For example, in Pichicolo, Quillaipe, and Canutillar during the spawning season from 2007 to 2008, up to three different sized larval cohorts have been observed in September, increasing to five different larval cohorts in December 2007 (Avendaño et al. 2011). *M. chilensis* in the Reloncaví zone typically have a seven month spawning season from September to March (Stotz 1981). Active spawning was observed from December/January to March in Quillaipe and Pichicolo by Avendaño et al. (2011) and seeds reach a size of 1–2 cm after 3 or 4 months after fixing (Clasing et al. 1998). This size is concordant with our sampling dates in June for Reloncaví locations (Larrain et al. 2012), suggesting that seeds collected in this zone came from the natural banks of these same locations. Thus, the signal of genetic differentiation within this region would be due to either neutral variation among cohorts or possibly adaptive variation associated with family traits in each cohort.

Outlier tests have been shown to have high false‐positive rates under certain study conditions, so candidate loci must be interpreted with caution (Lotterhos and Whitlock 2014). The *F*_ST_ outlier detection method FDIST2 used here (Beaumont and Nichols 1996) has proved to have good performance in species with low genetic differentiation and can be powerful at detecting adaptive variation relative to other outlier tests under certain scenarios (Narum and Hess 2011; De Mita et al. 2013; Lotterhos and Whitlock 2014). In our study, some *F*_ST_ outlier SNPs may be interpreted as genetic variation in response to adaptation to local environmental conditions (Beaumont 2005), but other alternative explanations are also possible (Gosset and Bierne 2013) such as the examples of different cohorts described above or other endogenous barriers (Bierne et al. 2011) and introgression (Fraïsse et al. 2015). *Mytilus* species are prone to produce hybrid in geographical areas where two sibling species coexist (Daguin et al. 2001; Toro et al. 2004a), given the opportunity to introgress alleles from one species into the other, producing higher *F*_ST_ when these loci are evaluated in the genome of the recipient species. Such processes have been shown to cause signals of outlier *F*_ST_ in two length polymorphism loci between Atlantic and Mediterranean populations of *M. galloprovincialis* (Gosset and Bierne 2013). Very large *F*_ST_ values ≥0.5 can be found when different species of *Mytilus* and their hybrids are compared, even when outlier loci are removed (Zbawicka et al. 2014), but this large genetic differentiation was not evident with the neutral or outlier SNP panels developed here. However, in light of a hybrid zone recently reported in the Magellan Strait (Oyarzún et al. 2016), introgression is still a possibility to explain high *F*_ST_ values reported here. This hybrid zone shows decreasing levels of hybridization from the Magellan Strait to the north and is far from Isla Peel, our southern sampling location.

In our study, both neutral and adaptive processes, and even introgression, may be contributing to high *F*_ST_ values for outlier loci. Regardless of the underlying processes affecting these loci, these outliers provide useful tools for assigning stocks of *M. chilensis* to region of origin.

### Traceability implications

Traceability of aquaculture products is necessary in order to effectively regulate poaching and overharvest of natural populations, maintain genetic diversity of propagated stocks (e.g., Ogden 2011) and for food quality and safety purposes (Larraín et al. 2014). For *M. chilensis*, traceability with genetic methods is an important goal in order to preserve natural stocks and improve aquaculture practices in this species. Previous studies with small numbers of genetic markers (nine microsatellites) have only achieved 50.7% correct re‐assignment to origin locations which is too low to be used effectively in traceability applications (Larraín et al. 2014). However, our current study with 1240 SNPs has demonstrated that various panels of these markers can strongly assign individuals to the three macrogeographical zones included in scenario 1 (~92% of overall correct assignment). Also it was possible to re‐assign individuals to the Chiloé Island location with relatively high performance (~87%) using the 34 SNP panel (scenario 2).

In conclusion, our study demonstrates that species with high gene flow may require analyses at different geographical scales and multiple panels of putative adaptive loci to reveal hidden population structure. The SNP marker panels developed here increased assignment accuracy compared to previous microsatellite loci, probably due to the use of adaptive loci and the number of markers employed. Genetic traceability for *M. chilensis* with these *F*_ST_ outlier SNP panels is much improved but it is necessary in future research, to test the effectiveness of these panels in a wider zone and across years to evaluate the temporal stability of allele frequencies.

## Conflict of Interest

None declared.

## Supporting information


**Data S1**. Name of SNP makers developed for Chilean mussel.
